# Next-generation sequencing in the diagnosis of viral encephalitis: sensitivity and clinical limitations

**DOI:** 10.1038/s41598-020-73156-3

**Published:** 2020-09-30

**Authors:** Karol Perlejewski, Iwona Bukowska-Ośko, Małgorzata Rydzanicz, Agnieszka Pawełczyk, Kamila Caraballo Cortѐs, Sylwia Osuch, Marcin Paciorek, Tomasz Dzieciątkowski, Marek Radkowski, Tomasz Laskus

**Affiliations:** 1 Department of Immunopathology of Infectious and Parasitic Diseases, Medical University of Warsaw, Warsaw, Poland; 2 Department of the Medical Genetics, Medical University of Warsaw, Warsaw, Poland; 3 Department of Adult Infectious Diseases, Medical University of Warsaw, Warsaw, Poland; 4 Department of Microbiology, Medical University of Warsaw, Warsaw, Poland

**Keywords:** Infectious-disease diagnostics, Viral infection

## Abstract

Identification of pathogens causing viral encephalitis remains challenging, and in over 50% of cases the etiologic factor remains undetermined. Next-generation sequencing (NGS) based metagenomics has been successfully used to detect novel and rare infections, but its value for routine diagnosis of encephalitis remains unclear. The aim of the present study was to determine the sensitivity of shotgun metagenomic sequencing protocols, which include preamplification, and testing it against cerebrospinal fluid (CSF) samples from encephalitis patients. For sensitivity testing HIV and HBV positive sera were serially diluted in CSF from an uninfected patient. NGS repeatedly detected HIV and HBV sequences present at concentrations from 10^5^ to 10^2^ and from 10^5^ to 10 viral copies/reaction, respectively. However, when the same protocols were applied to RT-PCR/PCR positive CSF samples from 6 patients with enteroviral encephalitis (median viral load 47 copies/ml) and 15 patients with HSV, CMV or VZV encephalitis (median viral load 148 copies/ml), only 7 (28.6%) were identified as positive. In conclusions, while NGS has the advantage of being able to identify a wide range of potential pathogens it seems to be less sensitive compared to the standard amplification-based assays in the diagnosis of encephalitis, where low viral loads are common.

## Introduction

Despite steady advances in diagnostics, identification of pathogens affecting the central nervous system (CNS) remains challenging and in more than 50% of encephalitis cases the etiologic factor remains undetermined^1,2^. Rapid and accurate identification of a causative pathogen is often essential for a timely and proper clinical intervention^3^. Diagnostics of CNS infections is complicated by the sheer number of potential pathogens including over a hundred viruses as well as a larger number of bacteria, fungi and parasites which are capable of infecting CNS^4, 5^. However, viruses are clearly the predominant etiological factor and a recent comprehensive epidemiological study found that they are responsible for up to 69% of all encephalitis cases in the USA^6^.

Identification of pathogens is routinely conducted using serology and/or amplification of viral genome by RT-PCR or PCR^4^. However, as testing for a large number of agents is costly and impractical, these are typically aimed at detection of the most common pathogens expected in a particular epidemiological setting^4, 7^. In 2015 the U.S. Food and Drug Administration (FDA) approved the first multiplex PCR system marketed by BioFire Diagnostics; USA which allows for detection of 14 different common pathogens but only seven viruses known to infect CNS are included in the testing panel^8^. Thus rare, emerging and novel viruses would remain undetected.

Given the current limitations, next-generation sequencing (NGS) based metagenomics seem to present a potential solution to the problem presented by multiple pathogen etiology^5,9^. Massively parallel sequencing can theoretically identify all potential pathogens in a single run^10^. Since viruses lack the presence of any universal molecular marker such as 16S rRNA gene in the case of bacteria, the only suitable NGS technique capable of identifying viral species with a high taxonomic resolution is shotgun metagenomics^11,12^. However, while the latter technique is very robust for identification of low copy number pathogens, which is typical for viral encephalitis, it requires depletion of human/bacterial genetic background and enrichment of the target template^13,14^.

The aim of the present study was to determine the sensitivity of our shotgun metagenomic sequencing protocols which include filtration, DNase treatment and preamplification steps for the detection of RNA and DNA viruses and testing it against a panel of well-defined cerebrospinal fluid (CSF) samples from encephalitis patients.

## Results

### Sensitivity of metagenomics for the detection of RNA and DNA viral template

Serial dilutions of HIV and HBV standards were prepared, sequenced and analyzed in two independent runs (Run A and B) and the results are presented in Table 1. Total numbers of reads in both runs were similar: 165,980,852 and 156,803,545. After quality control, adapter removal and trimming the average number of reads per sample was 11,552,731 and 10,991,219 for run A and B, respectively. HIV reads were detected in both runs in every dilution from 10^5^ to 10^2^ copies/reaction but not in the dilution containing 10 copies/reaction and the percentage of recovered genome was decreasing with lower template number (Table 1).

**Table 1:** Results of NGS-based metagenomic analysis of serial dilutions of HIV and HBV in CSF.

HIV	Viral copies per reaction	Raw reads	Reads after quality control	Human reads	Viral reads	% of viral reads	HIV reads	% of HIV reads	HIV genome recovery (%)
A	10^5^	14,425,524	13,836,833	422,674	19,528	0.141	13,728	0.09921	67
	10^4^	6,302,313	6,021,715	2,571,701	19,783	0.329	9391	0.15595	54
	10^3^	13,159,332	12,682,116	7,839,532	16,908	0.133	2238	0.01765	9
	500	11,944,049	11,690,730	6,172,552	8457	0.072	2964	0.02535	35
	10^2^	11,800,215	11,547,630	7,783,788	4117	0.036	577	0.00500	20
	10	11,726,925	11,509,277	3,927,044	1419	0.012	0	0	0
	Neg. Control	11,925,730	11,703,429	5,933,379	92,406	0.790	0	0	0
B	10^5^	11,668,142	11,298,061	105,299	10,011	0.089	8439	0.07469	67
	10^4^	12,840,081	12,627,847	6,785,084	12,693	0.101	7948	0.06294	47
	10^3^	12,861,679	12,473,671	4,356,706	10,317	0.083	1123	0.00900	30
	500	12,872,252	12,628,312	4,888,315	2379	0.019	1066	0.00844	39
	10^2^	11,159,645	10,935,932	3,228,197	25,837	0.236	3	0.00003	1
	10	10,414,976	10,181,921	5,392,287	10,443	0.103	0	0	0
	Neg. Control	12,879,989	12,600,758	4,406,577	1850	0.015	0	0	0

HBV	Viral copies per reaction	Raw reads	Reads after quality control	Human reads	Viral reads	% of viral reads	HBV reads	% of HBV reads	HBV genome recovery (%)
A	10^5^	10,360,518	10,191,944	5,609,095	2,699,594	26.488	2,698,950	26.48121	100
	10^4^	8,559,975	8,437,550	6,743,671	496,994	5.890	492,685	5.83920	100
	10^3^	9,742,744	9,534,711	3,898,027	19,002	0.199	18,321	0.19215	100
	500	9,792,131	9,662,284	6,819,675	7,808	0.081	6239	0.06457	100
	10^2^	8,438,163	8,372,981	6,155,310	880	0.011	535	0.00639	92
	10	13,666,844	13,330,185	12,882,343	759	0.006	31	0.00023	23
	Neg. Control	10,404,653	10,139,861	4,566,305	1,594	0.016	0	0	0
B	10^5^	13,274,157	12,611,093	5,767,513	1,735,930	13.765	1,725,534	13.68267	100
	10^4^	7,119,784	7,023,868	6,524,333	58,633	0.835	57,927	0.82472	100
	10^3^	11,917,699	11,536,761	4,637,699	11,253	0.098	9338	0.08094	100
	500	9,559,985	10,742,873	5,195,146	17,750	0.165	16,966	0.15793	100
	10^2^	12,690,433	12,156,037	7,501,753	3,027	0.025	2236	0.01839	100
	10	6,481,227	6,273,703	1,321,592	2,420	0.039	225	0.00359	45
	Neg. Control	11,063,496	10,786,232	8,356,602	413	0.004	0	0	0

Percent of viral/HIV/HBV was assessed as of total number of reads after quality control.

Sequencing of HBV serial dilutions provided 70,965,028 (mean per sample: 10,219,414) and 72,106,781 (mean per sample: 10,137,861) reads for run A and B, respectively (Table 1). HBV-specific reads were detected in all dilutions from 10^5^ to 10 copies/reaction in both independent runs and the percentage of recovered genome was 100% for all but the very last dilutions (Table 1). Importantly, neither HIV nor HBV sequences were detected in samples with no viral template input. However, 413 to 92,406 reads in these samples mapped to various other viruses present in viral genomic database (Table 1).

### Metagenomic detection of viral pathogens in CSF

The above described protocols were applied to CSF samples from 21 patients with encephalitis of well-defined viral origin. Samples from 6 patients with enteroviral (EV) encephalitis were analyzed by RNA-based metagenomics (samples R1–R6), whereas samples from the remaining 15 patients (13 had HSV encephalitis, one had CMV encephalitis and one had VZV encephalitis) were subjected to DNA metagenomic workflow (samples D1–D15). Viral loads ranged from 12 to 458 copies/ml (median: 47 copies/ml) for EV and from 74 to 344 copies/ml (median: 148 copies/ml) for the three different DNA-viruses (Table 2).

**Table 2:** NGS-based metagenomic analysis of CSF from 21 patients with viral encephalitis of known etiology. Viral load was detected by real-time PCR using DNA/RNA extracted from 250 µl of CSF^37,40,48,49^.

Preamplification	Pt. ID	Age/gender	CSF white cells (in 1 μl)	Viruses detected by PCR (copies/ml)	Raw reads	Reads after quality control	Human genome reads	Viral reads	% of all viral reads as of total number of reads	Phage/all species (%)	Reads of virus detected by PCR	% Of reads representing virus detected by PCR	% Of recovered genome
RNA	R1	20/M	792	EV 458	12,764,480	11,711,129	5,303,301	3,497	0.030	97.56	2,253	0.01924	18
	R2	36/F	15	EV 60	13,869,622	12,338,503	2,964,460	5,106	0.041	96.88	0	0	0
	R3	25/M	73	EV 35	11,710,615	10,945,951	6,916,845	2,964	0.027	89.29	0	0	0
	R4	24/M	4	EV 18	13,383,351	12,178,616	5,973,727	4,966	0.041	100.00	0	0	0
	R5	54/M	5	EV 12	11,286,014	10,929,920	6,655,987	1,210	0.011	96.49	0	0	0
	R6	34/M	73	EV 78	12,112,027	11,705,702	6,070,198	1,859	0.016	100.00	0	0	0
DNA	D1	41/F	91	CMV 126	11,058,852	10,113,506	8,120,484	2,445	0.024	97.96	0	0	0
	D2	38/M	96	HSV 310	9,982,986	9,829,737	6,605,811	11,052	0.112	97.78	317	0.00322	3
	D3	36/M	4	HSV 230	12,240,215	11,530,488	3,377,199	3,728	0.032	95.24	109	0.00095	1
	D4	25/M	4	HSV 148	12,809,626	11,566,190	7,586,822	3,405	0.029	96.77	0	0	0
	D5	29/F	5	HSV 200	11,638,010	10,667,169	4,516,064	7,979	0.075	96.00	567	0.00532	4
	D6	27/M	79	HSV 246	9,108,760	8,554,862	6,274,423	4,225	0.049	100.00	0	0	0
	D7	58/M	60	HSV 138	11,710,615	10,945,951	5,888,655	8,431	0.077	97.37	1,361	0.01243	8
	D8	62/M	28	HSV 74	10,973,469	10,377,678	5,586,816	7,574	0.073	96.36	0	0	0
	D9	79/F	43	HSV 150	11,834,679	11,022,843	8,971,431	4,244	0.039	98.18	133	0.00121	2
	D10	32/M	83	HSV 72	13,479,480	11,931,036	7,773,594	10,299	0.086	66.67	410	0.00344	5
	D11	38/F	17	HSV 344	13,319,078	11,825,786	9,845,299	2,067	0.017	36.36	0	0	0
	D12	28/M	15	HSV 152	24,703,090	22,931,599	18,509,212	3,698	0.016	28.57	0	0	0
	D13	56/F	203	VZV 30	12,949,814	11,902,708	9,483,250	4,485	0.038	20.00	0	0	0
	D14	36/M	1225	HSV 76	14,744,148	13,382,202	10,372,565	5,791	0.043	78.95	12	0.00009	< 1
	D15	59/M	4	HSV 144	10,658,840	9,988,477	5,284,010	4,913	0.049	50.00	0	0	0

Next-generation sequencing generated 266,337,771 reads overall with an average number of 12,682,751 reads per sample (Table 2). An average number of reads per sample was similar for RNA (12,521,018) and DNA (12,747,444) analysis and the mean number of reads mapping to viral genomic database was 4,949 reads per sample, ranging from 1,210 to 11,052 reads.

When six CSF samples containing EV (samples R1 to R6) were analyzed, metagenomics revealed the presence of the expected pathogen only in the sample which had the highest viral load of all samples. In this sample 2,253 reads mapped to EV and this allowed for the reconstruction of 18% of enterovirus A genome.

In the case of 15 CSF samples containing DNA viruses, metagenomics identified the right pathogen in seven (samples D2, D3, D5, D7, D9, D10 and D14); (Table 2). The number of viral reads ranged from 12 (1% of recovered genome) to 1,361 (8% of recovered genome). In sample D14, HSV reads were mapping to the same region of viral genome and therefore, this sample was considered to be NGS negative as it did not fulfill our initially established criteria for positivity.

## Discussion

NGS-based metagenomics is a promising new tool in the diagnostics of a wide range of pathogens^15^. It has already been successfully applied in respiratory and intestinal infections and several groups have used this approach to identify causative agents in CNS infections^16–20^.

In the present study we evaluated metagenomics for the detection of RNA and DNA viruses using serial dilutions of viral template as well as CSF samples from encephalitis patients. Two major problems in metagenomic analysis of CSF are host and bacterial genetic background and low concentration of viral particles in this compartment^13^. To mitigate the first problem host/bacterial cells were separated by of low speed centrifugation followed by filtration and then samples were digested with DNase to degrade circulating free DNA not protected by viral capsid or envelope^13,21–23^. CSF is a low biomass sample—for example HSV, which is the most frequently identified viral encephalitis pathogen, has an average load of only 100 copies/ml^24,25^ and in our samples the median HSV concentration was only 150 copies/ml. While commercial NGS library systems require as little as 1 ng to 100 ng of nucleic acid input, in our study the amount of extracted DNA/RNA was below the levels of detection for Qubit HS (high sensitivity) kit, which is 0.2 ng and 5 ng for DNA and RNA, respectively. To overcome the problem of insufficient nucleic acid input we utilized commercially available Ovation RNA-Seq V2 System and SeqPlex Enhanced DNA Amplification Kit.

To evaluate the sensitivity of our methods we prepared serial dilutions of HBV and HIV viral template in CSF collected from an uninfected patient. We selected HBV and HIV as these were not present in any of the studied patients, making cross-contamination unlikely and are not the subject of any research in our lab thus lowering the risk of amplicon contamination. For HIV serial dilutions a positive alignment to HIV genome was obtained for samples containing from 10^5^ to 10^2^ viral copies per reaction. While in run A 10^2^ viral copies allowed for the reconstruction of 20% of HIV genome, for run B it was only 1% suggesting a likely limit of detection. In analogous experiments with HIV spiked into CSF free matrix Schlaberg et al. found the sensitivity of metagenomics to be approximately 100 copies/ml^26^. Similar sensitivity was reported by Edridge et al.^27^ who used virus discovery cDNA amplified fragment length polymorphism—next-generation sequencing protocol (VIDICSA-NGS) for the detection of RNA viruses in CSF. The authors were able to detect HIV in a sample containing 1.07 × 10^2^ viral copies/ml. In a protocol designed for the detection of both RNA and DNA viruses and using serial dilutions of equine arteritis virus and phocine herpesvirus 1 spiked into influenza A virus positive samples (recreating clinical sample background in respiratory system infections) van Boheemen et al. found the limit of detection to be 50–250 viral copies/reaction^28^.

Using serial dilutions of HBV in CSF we identified HBV reads in all dilutions from 10^5^ to 10 copies/reaction, but not in the negative controls. The percentage of genome recovery was high even for samples containing as little as 10 HBV copies (23% and 45% for runs A and B, respectively). These values are much higher than for the corresponding dilutions of HIV which could, at least in part, be influenced by the fact that the HBV genome (3.2 kb) is almost three times smaller than the genome of HIV (~ 10 kb). Previously mentioned VIDICSA-NGS failed to detect DNA viruses in CSF samples with viral load ranging from 5.28 × 10^3^ to 1.62 × 10^7^ copies/ml, but detected VZV present at a concentration of 9.29 × 10^7^ DNA viral copies/ml^27^. However, in two other studies the sensitivity of metagenomics for the detection of DNA viruses was very similar to our findings. Schlaberg et al*.* using CMV spiked into CSF found the limit of detection to be 9.4 copies/ml, while sensitivity of 10 copies/ml was reported by Xia et al*.* in their case report in which metagenomics was applied to detect human polyomavirus 2 in CSF from a patient with progressive multifocal leukoencephalopathy^17,26^.

When CSF samples from six patients with EV encephalitis were analyzed, the EV genome was detected only in the sample with the highest viral load (458 copies/ml) and only 18% of enterovirus A genome could be reconstructed. EV was not detected in any of the samples in which the viral load was below 100 copies/ml. Among 15 clinical CSF samples from patients infected with DNA viruses (13 samples with HSV, one with CMV and one with VZV), six were found to contain reads aligning to HSV genome. In one sample (D14) all 12 HSV reads mapped to the same position on HSV genome, thus they did not meet our initially established criteria for positive pathogen detection. Taking into account the exclusion of the latter sample, metagenomics confirmed etiology in 23.8% of all analyzed cases which is a proportion similar to that reported by a tertiary diagnostics center where 29.3% of metagenomic findings matched the results of routine diagnostic tests conducted on CSF, blood samples, throat swabs, stool and tissue biopsy samples^29^. In another study Wilson et al. showed 42% compatibility between metagenomics and routine diagnostic tests in patients with CNS infection but it should by emphasized that the protocol allowed not only for the detection of viruses but also bacteria, fungi and parasites. Detection of Herpesviruses could be negatively affected by DNase treatment. In our previous study we found that while DNase treatment resulted in more than twofold decrease in the number of host-derived sequences and increased the number of bacterial and other sequences 30–50 times, it reduced the yield of HHV-1 four-fold and markedly lowered gene coverage when plotted to full-length HHV-1 reference sequence^30^. This sensitivity of HHV-1 to DNase treatment has been since confirmed by others^27^ and seems to be due to the fact that in cell-free clinical material DNA of Herpesviruses is largely present in highly fragmented naked form and not as encapsulated virions^31^. In the study by Hong et al.^32^ which did not use the DNase digestion step, metagenomics detected HHV-1 in 5 out of 7 RT-PCR positive patients.

In addition to specific viral targets, multiple reads mapping to various viral genomes but primarily to bacteriophages were present in all analyzed samples, including negative controls. It was previously reported that bacterial DNA contamination of commercial DNA extraction kits and PCR reagents is very common and this is likely to be an indirect source of bacteriophagial genomes^30,33,34^. Since similar sets of viral sequences were present in multiple analyzed samples it is highly likely that they indeed represented such a contamination originating from reagents, although some could be the products of amplification errors^35,36^.

In conclusion, while NGS has the advantage of being able to identify a wide range of potential pathogens, its sensitivity in the diagnosis of viral encephalitis is still inferior to standard amplification-based assays.

## Methods

### Control and patient samples

The sensitivity of our DNA and RNA workflows was evaluated using human immunodeficiency virus type 1 (HIV; viremia 10^6^ copies/ml) and Hepatitis B virus (HBV; viremia 7 × 10^4^ copies/ml) positive sera, which were diluted in CSF from an uninfected patient. Final concentrations were adjusted to contain 10^5^, 10^4^, 10^3^, 500, 100 and 10 viral copies per reaction.

Next, our protocols were tested against a panel of well-defined 21 CSF samples from patients with encephalitis who were part of a large prospective epidemiological study of encephalitis in Poland^2^. Six patients had enteroviral infection, 13 had herpes simplex virus (HSV), one had cytomegalovirus (CMV), and one had varicella zoster virus (VZV). We tested all patients who were CSF-positive by real-time RT-PCR/PCR and in whom an unthawed vial of CSF sample was preserved from the original study. CSF samples were analyzed using in-house quantitative real-time RT-PCR/PCR described previously^37–40^.

### Nucleic acids extraction

After collection all CSF samples were centrifuged at 1200 rpm for 20 min at 4 °C, aliquoted and kept frozen at − 80 °C until analysis. Each 225 µl of CSF supernatant/standard was filtrated using Millex-HV Syringe Filter Unit (Merck KgaA, Germany) with a pore size of 0.45 μm and digested with 2U of TURBO DNase (Thermo Fisher Scientific, USA) for 30 min.

Next, 250 μl of filtrated and digested CSF/standard were subjected to RNA extraction with TRIzol LS (Thermo Fisher Scientific, USA) or DNA extraction using NucleoSpin Plasma XS kit (Macherey–Nagel, Germany), following manufacturers' protocols. RNA and DNA were eluted in 5 μl and 12 μl of water, respectively.

### RNA and DNA preamplification

Since the typical yield of RNA and DNA extraction from CSF was very low and below the limit of detection by Qubit dsDNA (> 0.2 ng) and RNA (> 5 ng) HS Assays (Thermo Scientific, USA), all samples and all standards underwent preamaplification. Five microliters of RNA was first reversely transcribed for 5 min at 65 °C and preamplified by a single-primer isothermal amplification (Ribo-SPIA)^41^ using Ovation RNA-Seq V2 system (NuGEN, San Carlos, USA) following manufacturer’s protocol. Preamplification of DNA was done using SeqPlex Enhanced DNA Amplification protocol (Sigma-Aldrich, USA); 12 μl of extracted DNA was loaded into each reaction which underwent 29 cycles of amplification. Preamplified cDNA and DNA were subsequently purified using 0.8 ratio of Agencourt AMPure XP beads (Beckman Coulter, USA) to reaction mixture and finally eluted in 30 μl of water. To assess the ability of pre-amplification steps to enrich RNA and DNA input, we spiked known number of copies of either RNA (represented by HIV) or DNA (represented by HBV) virus into negative CSF sample and performed filtration, nuclease digestion and RNA/DNA extraction steps followed by either RNA/DNA pre-amplification or no pre-amplification. Real-time PCR revealed that the preamplification step increased the yield of both RNA and DNA viral genomes significantly (Supplementary Information).

### Library preparation and sequencing

Libraries for sequencing were prepared by Nextera XT Kit (Illumina, USA) using one ng of preamplified cDNA/DNA and following manufacturer’s protocol with two minor modifications: the number of amplification cycles was increased from 12 to 14 cycles and the ratio of Agencourt AMPure XP beads (Beckman Coulter, USA) to reaction mixture in the last cleanup step was 0.6. The quality and average length of NGS libraries were assessed using Bioanalyzer (Agilent Technologies, USA) and DNA HS kit (Agilent Technologies, USA). Next, samples were indexed, pooled and sequenced on Illumina HiSeq (101nt, paired-end reads).

### Next-generation sequencing (NGS) data analysis

Reads generated in NGS were evaluated for their quality using FastQC (Phred quality score above 30)^42^. Adapter removal and trimming were done with the help of Trimmomatic software^43^. All filtered reads were first mapped to human reference sequence (hg19) using Stampy^44^ and the remaining, unmapped reads were aligned to NCBI RefSeq viral genomic database (9238 complete viral genomes) by Bowtie2^45^. All viral reads were sorted and counted with SAMtools and phyloseq package in R^46,47^. Visualization of alignments, coverage, and calculations of percentage of recovered genomes were done using CLC Genomics Workbench (Qiagen, Germany).

The following criteria were applied for positive virus detection by NGS: (1) at least three reads specific for a particular viral species, (2) reads distributed over the whole genome, (3) no presence of any of the former viral reads in the negative control samples. The same criteria were previously applied by other groups for NGS identification of viruses^26,29^.

All patients gave a written informed consent and all methods were performed in accordance with the relevant guidelines and regulations. The study was approved by the Internal Review Board of Warsaw Medical University.

## Supplementary information

Supplementary Information.

Supplementary Table.

## Data Availability

The datasets generated during the current study are available in the Sequence Read Archive (SRA accession: PRJNA658239).
