# Supplementary material for: Next-generation sequencing in the diagnosis of viral encephalitis: sensitivity and clinical limitations
Source: Sci Rep. 2020 Sep 30;10:16173. doi: 10.1038/s41598-020-73156-3 (PMC7528011; doi:10.1038/s41598-020-73156-3)
Supplement: Supplementary file 1 — Supplementary Information. [file 41598_2020_73156_MOESM1_ESM.docx]

**Next-generation sequencing in the diagnosis of viral encephalitis: sensitivity and clinical limitations**

Karol Perlejewski*^1^, Iwona Bukowska-Ośko^1^, Małgorzata Rydzanicz^2^, Agnieszka Pawełczyk^1^, Kamila Caraballo Cortѐs^1^, Sylwia Osuch^1^, Marcin Paciorek^3^, Tomasz Dzieciątkowski^4^, Marek Radkowski^1^, Tomasz Laskus^3^

^1^ Department of Immunopathology of Infectious and Parasitic Diseases, Warsaw Medical University, Warsaw, Poland;

^2^ Department of the Medical Genetics, Warsaw Medical University, Warsaw, Poland;

^3^ Municipal Hospital for Infectious Diseases, Medical University of Warsaw, Warsaw, Poland.

^4^ Department of Microbiology, Warsaw Medical University, Warsaw, Poland.

Corresponding author*

Karol Perlejewski, PhD

Department of Immunopathology of Infectious and Parasitic Diseases

Medical University of Warsaw

3C Pawińskiego Street, 02-106 Warsaw, Poland

kperlejewski@wum.edu.pl

**Methods:**

The assess the effect of preamplificatiton steps on viral template, serum containing human immunodeficiency virus type 1 (HIV; viremia 10^6^ copies/ml) and serum containing Hepatitis B virus (HBV; viremia 7x10^4^ copies/ml) were diluted in CSF from an uninfected patient to a final concentration of 10^4^ viral copies per reaction. For each sample 225 µl was filtrated using Millex-HV Syringe Filter Unit (Merck KgaA, Germany) with a pore size of 0.45 μm and digested with 2U of TURBO DNase (Thermo Fisher Scientific, USA) for 30 min. Next, filtrated and digested template standards were subjected to RNA extraction with TRIzol LS (Thermo Fisher Scientific, USA) or DNA extraction using NucleoSpin Plasma XS kit (Macherey-Nagel, Germany), following manufacturers protocols. HIV standards were either reversely transcribed with random hexamers and 200 units of M-MLV RT (Thermo Fisher Scientific, USA) or were subjected to single-primer isothermal amplification (Ribo-SPIA) using Ovation RNA-Seq V2 system (NuGEN, San Carlos, USA) following manufacturer’s protocol. In the next step 2ul of hexamers reverse transcription and RIBO-SPIA reactions, respectively, were subjected to real-time PCR using LightCycler FastStart DNA Master SYBR Green (Roche, Switzerland) containing antisense primer 5′CTGAAGGGTACTAGTAGTTCCTGCTATGTCACTT3′; nucleotides (nt 1488-1521) and sense primer 5′GGACATCAAGCAGCCATGCAAATGTT3′ (nt 1366 to 1391).

Preamplification of HBV standard was done using SeqPlex Enhanced DNA Amplification protocol (Sigma-Aldrich, USA) and both preamplified and non-amplified standards were subjected to real-time PCR using LightCycler FastStart DNA Master SYBR Green (Roche, Switzerland) containing antisense primer 5’CAAGGTATGTTGCCCGTTTG3' (nt 329-348) and sense primer 5’AAAGCCCTACGAACCACTGA3' (nt 587-568),

As shown in Table S1, DNA and RNA preamplification increased the yield 133,826 and 729-fold, respectively.
