# Supplementary material for: Next-generation sequencing in the diagnosis of viral encephalitis: sensitivity and clinical limitations
Source: Sci Rep. 2020 Sep 30;10:16173. doi: 10.1038/s41598-020-73156-3 (PMC7528011; doi:10.1038/s41598-020-73156-3)
Supplement: Supplementary file 2 — Supplementary Table. [file 41598_2020_73156_MOESM2_ESM.docx]

**Next-generation sequencing in the diagnosis of viral encephalitis: sensitivity and clinical limitations**

Karol Perlejewski*^1^, Iwona Bukowska-Ośko^1^, Małgorzata Rydzanicz^2^, Agnieszka Pawełczyk^1^, Kamila Caraballo Cortѐs^1^, Sylwia Osuch^1^, Marcin Paciorek^3^, Tomasz Dzieciątkowski^4^, Marek Radkowski^1^, Tomasz Laskus^3^

^1^ Department of Immunopathology of Infectious and Parasitic Diseases, Warsaw Medical University, Warsaw, Poland;

^2^ Department of the Medical Genetics, Warsaw Medical University, Warsaw, Poland;

^3^ Municipal Hospital for Infectious Diseases, Medical University of Warsaw, Warsaw, Poland.

^4^ Department of Microbiology, Warsaw Medical University, Warsaw, Poland.

Corresponding author*

Karol Perlejewski, PhD

Department of Immunopathology of Infectious and Parasitic Diseases

Medical University of Warsaw

3C Pawińskiego Street, 02-106 Warsaw, Poland

kperlejewski@wum.edu.pl

**Table S1. The effect of RNA/DNA preamplification on Real-time PCR amplification of HBV and HIV template**

|  | **Viral copies per reaction** | **Ct_(no preamp.)_** | **Ct_(with preamp.)_** | **Template increase**  **(x times)** |
| --- | --- | --- | --- | --- |
| **HBV** | **10^4^** | 27.82 | 10.79 | ≈ 133,826 |
| **HIV** | **10^4^** | 34.34 | 24,83 | ≈ 729 |
